# Impact and copying mechanisms towards retakes: A case study of five health training institutions in Sub Saharan Africa

**DOI:** 10.21203/rs.3.rs-5374432/v1

**Published:** 2024-12-16

**Authors:** RONALD KIBUUKA, Richard Katuramu, Samuel Owusu-Sekyere, Irene Atuhairwe, Brian Agaba, Prossy Nakattudde, Abigail Amponsah, Ndikom Chizoma, Ogah Oluwakemi, Kennedy Kiyimba, Samuel Baker Obakiro, Atipasta Kaminga, Joshua Epuitai, Enid Kagoya Kawala, Etta Chimbe, Masumbuko Baluwa, Getrude Munthali, Getrude. Tamala Phiri, Dan Kibuuke, Ferastas Mpasa

**Affiliations:** Busitema University Faculty of Health Sciences Department of Pharmacology and Therapeutics; Busitema University Faculty of Health Sciences Department of Internal Medicine; African Forum for Research and Education in Health; Seed Global Health; Seed Global Health; Seed Global Health; Kwame Nkrumah University of Science and Technology; University of Ibadan; University of Zambia School of Medicine; Busitema University Faculty of Health Sciences Department of Pharmacology and Therapeutics; Busitema University Faculty of Health Sciences Department of Pharmacology and Therapeutics; Mzuzu University; Busitema University Faculty of Health Sciences Department of Nursing; Busitema University Faculty of Health Sciences Department of Public Health; Mzuzu University; Mzuzu University; Mzuzu University; Mzuzu University; Busitema University Faculty of Health Sciences Department of Pharmacology and Therapeutics; Mzuzu University

**Keywords:** retakes, health professions education, Sub-Saharan Africa, OSCE

## Abstract

**Background:**

Academic examination retakes are significant challenges in health professions education. With rigorous clinical assessments and high-stakes examinations, many students struggle to meet academic requirements, resulting in retakes. The voices and experiences of such students have often been absent within the broader discussion of health professions education. This study aimed to assess the impact and copying mechanisms of medical and nursing students with retakes across five Sub-Saharan African universities.

**Methods:**

This was a mixed-methods explanatory sequential study, integrating quantitative surveys and qualitative interviews. The study involved 764 medical and nursing clinical students from five universities across Sub Saharan Africa: Busitema University (Uganda), Mzuzu University (Malawi), University of Ibadan (Nigeria), Kwame Nkrumah University of Science and Technology (Ghana), and the University of Zambia. Quantitative data were analyzed using descriptive statistics. Qualitative data were explored through thematic analysis based on in-depth interviews with 15 purposively selected students who had experienced retakes.

**Results:**

Overall, the proportion of students who had ever had a retake in clinical assessments was 12.6%. Thematic analysis revealed emotional and psychological challenges, such as shock, anxiety, fear and suicidal thoughts particularly due to insufficient institutional support and lack of feedback. Many students also reported a perceived sense of unfairness in their assessments.

**Conclusions:**

The study highlights the multifaceted nature of retakes in health professions education in Sub-Saharan Africa. Recommendations for improvement include providing detailed feedback, transparency in assessments, and enhancing both academic and psychological support systems for students, particularly those at higher risk, such as older students and males. Further research is needed to investigate long-term academic and career outcomes and effective remediation strategies for students experiencing retakes.

## Introduction

The journey through medical and nursing education is indeed fraught with challenges, especially during the clinical years, where students face high-stakes examinations and the transition from preclinical to clinical training ^1^. The clinical years of health professions education are often associated with a notable incidence of retakes. Studies have shown that the average proportions of students getting retakes in undergraduate health professions education is around 10%, with a noted 30–50% of all medical students having ever experienced stress ^2,3^. From the Busitema University academic handbook 2020/21–2023/24 a retake is defined as the act of a student repeating a course or courses when next offered to achieve the pass mark (50%) if they failed during the first assessment or to improve a low pass grade, with the original grade being retained on the transcript if no improvement is made ^4^. However, in this study a retake only refers to a student failing to achieve the pass mark (50%). Performance can be affected by the pressure to perform well in high-stakes examinations. Clinical evaluations can lead to increased stress and anxiety, making it challenging for students to maintain their motivation and self-confidence ^5^, this can be worsened in case a student gets a retake.

Research indicates that medical education is inherently stressful due to various factors, including demanding clinical rotations, rigorous assessments, and the pressure to maintain professional standards ^6^. Studies have consistently shown that health professions students experience high levels of stress, which can negatively impact their academic performance and overall well-being ^7–9^. Studies byAryaei Moghadam et al., 2023a andPrendergast et al., 2024 highlight that many medical students experience high levels of stress and burnout, which can detrimentally affect their academic performance. Furthermore,Pasic et al., 2020 andCampbell et al., 2022 identify personal issues and mental health challenges as significant contributors to academic difficulties and retakes among students.

The literature further reveals that the retakes in medical education can be attributed to various factors, including inadequate preparation, personal challenges, and mental health issues. A study at a UK university suggests that approximately 10–15% of medical students may fail to progress satisfactorily, which can have long-term implications for their professional practice ^14^. A retake can significantly impact students’ academic paths and mental well-being. Moreover, the traditional remediation strategies often focus on merely passing assessments rather than addressing the underlying issues that lead to retakes. This approach has been criticized for potentially perpetuating the cycle of retakes rather than fostering genuine understanding and improvement ^14,15^.

Even though students who experience retakes face academic challenges, the voices and experiences of such students have often been absent within the broader discussion of health professions education. These students are capable of commenting profusely on their failures and probably can shed more light on both systemic and personal factors contributing to their academic difficulties. It is important to note that these experiences reflect not only on the fairness and effectiveness of the current assessment methods but also on the emotional health of the students who have to go through the pain of retakes. These voices will improve support systems and interventions given to the students for better academic outcomes and welfare.

## Methodology

### Study Design

We used a mixed-methods sequential explanatory research design that involved both quantitative and qualitative methods. The quantitative study was a cross-sectional design while the qualitative study involved in-depth interviews.

### Study Setting and Participants

The study was conducted across five universities in sub–Saharan Africa: Busitema University (Uganda), Mzuzu University (Malawi), University of Ibadan (Nigeria), Kwame Nkrumah University of Science and Technology (Ghana), and the University of Zambia (Zambia).

#### Mzuzu University, commonly Known as MZUNI, Malawi:

Chartered in 1997 under Chap. 30:09 of the Laws of Malawi, Mzuzu University is a public institution of higher learning committed to fostering knowledge and offering quality education, research, and training. The university aims to meet the educational expectations for Malawi, Africa, and the global community. Courses offered include, from Certificate to Doctorate, and are divided into six faculties that encompass Health Sciences, Education, and Environmental Sciences. It is a dual-mode institution offering face-to-face and Open Distance e-Learning. In addition, over 50 research projects have so far been successfully executed within the university with major funding from international organizations such as the World Bank, ACIAR, and USAID. These projects include, among others, ICT, climate change, health, and renewable energy studies.

### Busitema University, Uganda

Busitema University is a multi-campus public university in Eastern Uganda. It was established in 2007 by Statutory Instrument No. 22 under the Universities and Other Tertiary Institutions Act. Its mission is to provide high-standard training and engage in research that drives socio-economic transformation and sustainable development. Busitema University holds a commitment to academic excellence as it contributes towards the greater development activities of Uganda through research and community outreach.

**The University of Ibadan, Nigeria** was established in 1948 initially as a college of the University of London, then becoming a full-fledged independent university in 1962. Largely recognized as Nigeria’s premier university and its first, the mission of the university is “to expand the frontiers of knowledge through academic excellence geared towards meeting the needs of society.”. UI’s College of Medicine is the first in Nigeria and was established in 1948, followed by the pioneering Nursing program in 1965.

### Kwame Nkrumah University of Science and Technology, Ghana

KNUST was founded in 1951 as the Kumasi College of Technology and became a full-fledged university in 1952; it was later renamed Kwame Nkrumah University of Science and Technology. The vision for this university is that it should be among the leading science and technology institutions in Africa, driving the creation and utilization of knowledge through research, entailing quality teaching and learning, and community involvement through outreach. KNUST commits itself to the promotion of innovation, entrepreneurship, and technological leadership in all its academic and research programs.

### University of Zambia, Zambia

The University of Zambia was established in 1965 and is the largest and oldest learning institution in Zambia. The University officially opened its doors in 1966. UNZA provides education through the medium of English and lies at the heart of Zambia’s education and research. It is one of the most important universities, particularly in training in the areas of health and medical sciences, in furthering academic development both nationally and regionally.

The study sites were selected purposively because they were part of the joint-funded project by AFREhealth small grants Cohort 2 (2023) and also ensuring African regional representation. They have different faculties which include Faculty of engineering, faculty of biological sciences, faculty of education, faculty of agriculture and faculty of health sciences. We then chose students who were in the faculty of health sciences offering Bachelor of medicine and Bachelor of Surgery and Bachelor of Nursing

### Quantitative Component:

#### Study population

The study population consisted of final year clinical healthcare professionals. Students offering a bachelor of medicine and surgery and bachelor of science in nursing were included in the study. Final year students were selected because they had been exposed to several clinical assessment formats multiple times. This ensured that they had sufficient experience with clinical assessments to provide meaningful insights.

The qualitative component of this study focused on a subset of students who had experienced academic retakes during their final year of medical (MBChB) or nursing programs across the five participating universities.

### Sampling method

#### Estimation of Sample Size

Since the target population is finite, the following formula (Krejcie & Morgan, 1970) was used to determine the sample size ^16^. This formula provides a systematic way of determining the appropriate sample size in relation to the total population to ensure an adequate level of precision in statistical analysis. The formula takes into consideration population size, desired confidence level, and margin of error. Using this approach, the total sample size was determined to be 571 students drawn from the following universities:

Finite population: $$CI^{\prime }=\hat{p}\pm \text{z}\times \sqrt{\frac{\hat{p}\left(1-\hat{p}\right)}{n^{\prime }}}\times \frac{N-n^{\prime }}{N-1}$$

Where:

z is z score

$\hat{\text{p}}$ is the population proportion

n and ${\text{n}}^{\prime }$ are sample size

N is the population size

The final sample size for this study was 571 students, as shown in the Table 1 below:

Sampling for the in-depth interviews

For the qualitative component, purposive sampling was used to select participants for in-depth interviews. This sampling method ensured that students who had first-hand experience with academic retakes during their final year in medical (MBChB) or nursing programs were included. A total of 15 students—three from each of the five participating institutions (Busitema University, Mzuzu University, University of Ibadan, Kwame Nkrumah University of Science and Technology, and the University of Zambia) were selected. Three were selected because the institution with the lowest occurrence of retakes had three students so to ensure balance of participants three studnts were chosen from each institution

The selection aimed to ensure diversity across key demographics such as gender, age, and program of study (nursing and medicine). This approach allowed for a rich exploration of the personal and emotional experiences of students who had encountered retakes, as well as their perceptions of the systemic and institutional challenges that contributed to their academic difficulties. The purposive sampling ensured that the most relevant participants were included, as they could provide in-depth insights into the factors influencing academic retakes, including both personal and institutional aspects. The smaller sample size for the qualitative component allowed for a deeper exploration of individual experiences and perceptions.

### Study procedure

A standardized questionnaire was self-administered to 764 participants in order to collect data on demographics and any history of retakes during clinical years. Those who had ever experienced retakes and were willing to take part in the in-depth interviews were asked to submit their WhatsApp phone numbers. The instrument was shared online via email and institutional platforms, and responses were collected over four weeks. This was then followed by a WhatsApp phone call after appointments and consent to participate in the study through WhatsApp messaging

### Data collection for the in depth for the in depth interviews

In-depth, semi-structured interviews were conducted with a subset of 15 (3 from each institution) purposively selected students from different institutions who had experienced retakes. Participants were selected using purposive sampling, ensuring diversity in gender, program, and university. The interview guide included open-ended questions focusing on students’ experiences of failure, emotional and psychological impacts, perceived systemic challenges, coping mechanisms, and suggestions for improving the academic support system. Interviews were conducted via WhatsApp audio calls; WhatsApp is an online messaging and communication platform. These were then recorded with participants’ consent, and transcribed verbatim.

### Statistical analysis

The quantitative data were analyzed using descriptive statistics to summarize the demographic characteristics of the participants. Retake were calculated as percentages. All analyses were performed using SPSS version 25. After getting results of retake occurrences then a qualitative analysis followed to get a deeper understanding on physical and psychological factors affecting students with retakes plus the copying mechanisms and what the students believed could be the solutions to enable a smooth retake process.

Qualitative data analysis was done through thematic analysis. First, two independent researchers coded the transcripts to ensure inter-rater reliability. The codes were then grouped into higher-order themes such as emotional and psychological impact, systemic challenges, coping strategies, and institutional support. The Braun and Clarke six-phase framework guided this analysis: becoming familiar with the data, generating initial codes, searching for themes, reviewing themes, defining and naming themes, and writing the report ^17^. Atla ti 9 was used for data management and coding.

### Ethical Considerations

The study received ethical approval from Busitema University Research ethics committee BUFHS-2023-132 and administrative clearance from the institutional review boards of the participating universities. Prior to commencement of the study, a memorandum of understanding was signed by the participating institutions. Informed consent was obtained from all participants, ensuring confidentiality and the right to withdraw from the study at any time. All survey responses and interview transcripts were anonymized to protect participants’ identities.

## RESULTS

We approached 851 students and recruited 764 students. As shown in Table 2 below. A total of 764 participants were included in the study. More than half of the participants were female (61.9%). Majority of the participants were Christians (90.4%), followed by Muslims (5.8%). Participants’ ages ranged from 18 to 48 years, with a mean age of 25.38 ± 5.00 years. over a half of the participants (54.5%) were aged 18–24 years. The largest proportions of participants were enrolled in nursing programs (64.5%), and a significant majority (65.4%) entered their programs through direct entry. All participants were pursuing bachelor’s degrees.

Table 3 summarize the proportion of students who reported having a retake during clinical evaluation. The data shows significant differences across the institutions, with Mzuzu University and the University of Zambia having higher rates of students who had retakes compared to other universities. The overall failure rate was 12.6%, but the rates varied significantly by institution (p < 0.001). Kwame Nkrumah University had the lowest failure rate (2.5%), while Mzuzu University reported the highest failure rate (22.1%). Students at Busitema University and the University of Zambia also reported relatively high failure rates at 12% and 14.7%, respectively.

## Results (Qualitative Finding)

### Qualitative Results

This section presents the qualitative findings derived from in-depth interviews conducted from 15 (3 from each institution) purposively sampled final-year students from different Universities across Sub Saharan Africa who had experienced retakes. The analysis revealed several prominent themes: emotional and psychological impact, systemic challenges and perceived unfairness, coping mechanisms, impact on self-perception and motivation, support systems, and suggestions for improvement. Each theme is supported by rich narratives, highlighting the complex experiences of students facing retakes in health professions education.

**Table:** 

		Freq	Percentage
	Below 25	4	26.7%
age	25–34	9	60%
	35 and above	2	13.3%
Gender	Male	9	60%
	Female	6	40%
Program	MBChB	7	47%
	Nursing	8	53%

### 1. Emotional and Psychological Imapct

Failing an assessment had a profound **emotional and psychological impact** on students, with many describing their initial reactions as being filled with **shock, fear, anxiety and suicidal thoughts**.

One participant vividly described the emotional burden: “*…when I saw my marks, I couldn’t believe I had failed. It was shocking and overwhelming…*” (38 year male MBChB student from uganda) another student exclaimed *“…it’s hard. Yeah…It’s hard…I knew that this is going to be on my transcript forever nomatter what I do…” (*25 year male MBChB student from uganda). some students had to leave the school environment for a while *“…went to the village, because I just didn’t feel well…”* 25 year female Nursing student from Uganda and some students felt suicidal thoughts as noted by one student *“…I was devastated and suicidal… “*(30 year male MBChB student from Zambia)

The **fear of failure** was further compounded by the potential financial consequences and the fear of failing again, particularly for students on government sponsorships. One student explained how the prospect of failing a second time created immense pressure:

“…*If I fail this second time, it means I need to go on self-sponsorship… which I cannot afford.. I therefore worked harder .*” (25 year male MBChB student from uganda) another student also said *“…I felt bad, but I was also afraid that even if they gave me another chance, like am I going to make it?”* (26 year male MBChB student from Ghana)

While some students reported feeling **devastated** by the failure, others exhibited **resilience** and **acceptance** of their circumstances.

One participant emphasized the importance of adapting quickly: “…*I admit that I failed, and I don’t dwell on it. I quickly moved on to prepare for the next attempt…hoewever, I felt like it was a punishment* “ (28 year male Nursing student from Malawi) Another student noted that retaking is not a good experience “*…It’s not a good experience anyway. Anyone would want to pass at once…and giving a retake is something that can give you pressure* (30 year male Nursing student from Malawi). But some students noted the need for extra effort with confidence that they can make it *“…need for us to work hard. And to have that high self-esteem that I can do it.”* (21 year female Nursing student from Nigeria)

This acceptance was a coping mechanism for many, allowing them to refocus on their studies and reduce the emotional toll of the failure.

### 2. Challenges

Students faced systemic challenges and perceived the retakes as unfair. Several participants highlighted **systemic challenges** in the assessment process, particularly during the Objective Structured Clinical Examination (OSCE). Students frequently reported experiencing **unfair treatment** and **bias** from assessors.

One student described this discrepancy in the behaviors of different assessors “… *OSCE is unpredictable; sometimes assessors aren’t welcoming, and they rush you before you even finish reading the question…*” (21 year female Nursing student from Nigeria)

In addition, students expressed concern about **personal biases** among examiners. Relationships between students and lecturers often influenced the assessment outcomes, as noted by one participant “…*if you’re not in good terms with a lecturer, they might give you lower marks than you deserve…*” (26 year male Nursing student from Malawi)

However, some students noted out that failure can be attributed to their inadequate preparation for the assessment and low academic content. *“…Maybe it’s not studying enough… not following the schedules during evaluation or clinical testing.”* (25 year female Nursing student from Uganda) Another respondent further emphasized this “*…because if you fail to prepare, honestly, it takes out your confidence. So, that can also cause someone to fail…”* (23 year female nursing student from Nigeria)

Another major concern was the **lack of feedback** after failing an assessment. Many students reported that they were not informed of the specific areas in which they had underperformed, making it difficult to prepare effectively for the retake. One participant expressed frustration:

“… *to retake an assessment, I’m not always sure where I went wrong because we don’t get detailed feedback…*” *feedback is not given to us all you hear is you failed OSCE* (30 year male MBChB student from Zambia)

*“…they didn’t give me the areas that I failed… I’m not sure of where I’m retaking…”* (39 year male nursing student from Ghana) reechoed by another student.

In addition, students emphasized that the need to **repeat the whole course unit or the whole year** after failing one part of the assessment was unfair, one respondent highlighted, “…*if you fail the OSCE exam, you are not given another chance like immediately. You are taught to repeat the course itself, the whole course, including the theory, the practice…”* (29 year female MBChB student from Zambia) Another respondent noted *“…when you fail the practical part, it means you’re supposed to repeat the whole year… So, you’re looking at the expenses for the whole year..*.” (23 year female nursing student from Nigeria)

This lack of transparency left students feeling unprepared and uncertain about how to improve their performance.

Students finally noted out the system as a factor towards retakes, one student said; *“…the mere fact that the system allows for existence of retakes, increases the chances of people getting the retakes. If the system was shaped in a way that there is nothing like a retake, we would not be having retakes.”* 28 year male Nursing student from Malawi

### 3. Coping Mechanisms

Despite the challenges, students employed a variety of **coping mechanisms** to manage the stress of retakes. **Acceptance** of failure and a **focus on improvement** were common strategies. Other students noted getting involves in some activities such as music.

Students explained how they coped by quickly moving on “*…once I knew I had to retake, I started focusing on what went wrong and how I can improve… I didn’t let it pull me down…*” (26 year male Nursing student from Malawi) *“…I just believed in myself, and accepted that everything happens for a reason…”* (30 year male Nursing student from Malawi) Another student noted *“…I would take coffee mostly… play piano and teach music …”* (31 year male MBChB student from Zambia)

**Peer and parental support** also played a crucial role in helping students navigate the retake process. Many participants mentioned that they relied heavily on their friends and parents for guidance and encouragement.

Students shared:

“…*I relied heavily on my friends to help me prepare for the retake; they were the ones who encouraged me and guided me in areas I wasn’t sure about…*” (38 year male MBChB student from uganda) *“…my parents supported me that this is another chance… my friends were there for me…* (21 year female Nursing student from Nigeria)

However, while peer support was readily available, **institutional support** was often lacking. Students frequently expressed frustration that they did not receive guidance from lecturers or academic advisors, which made many feel like the retake was a punishment. One student lamented:

“…*There’s not enough time to seek advice from lecturers, and I didn’t receive any guidance from academic advisors…*” (26 year male Nursing student from Malawi)

This gap in formal support left many students feeling isolated in their preparation for retakes.

### 4. Impact on Self-Perception and Motivation

The experience of failing an assessment had a lasting impact on students’ **self-perception** and **motivation**. Many participants reported a significant decline in their confidence and academic performance following a retake.

Student reflected on how their failure affected their academic standing: *“…I felt like I got nowhere in my studies. I’m not teachable and I can’t even succeed…”* (31 year male MBChB student from Zambia) students also noted *“… it really affected me. Because previously, I felt like I was a bright student. And I didn’t see myself repeating any course or having a low grade… I was doing well…the time I started to retake this class, this course…my grades went down for that year…I had that feeling of frustration and anxiety…it really affected my grade… I saw it even with my grade, it went down…motivation to work hard went down…”* (38 year male MBChB student from uganda), *“…I used to participate in class. I used to work hard… but honestly, I stopped. Because I saw no reason of working hard… just to graduate…”* (39 year male nursing student from Ghana) Further respondents reported an impact on their self-esteem *“…when you fail… you have that low self-esteem… like you have failed a thing that you already know, a thing that you have been practicing on the ward. So, it’s like you have that low self-esteem”* (38 year male MBChB student from uganda) another student also noted *“…when you’re retaking those exams, you will feel like you are the worst students for the rest of the class. You feel like you are incompetent because at the end of the day, if you are given a retake, it means you are the worst students. So, some could laugh, some could talk behind you, but at the end of the day, you just need to move on…”* (22 year female Nursing student from Nigeria)

For some students, however, the retake experience served as an opportunity for **academic growth**. Some students explained how they used the retake to strengthen their understanding of the material:

“…*The retake experience opened me up to deeper understanding of concepts I had struggled with before… I became the student I always wanted to be.*” (31 year male MBChB student from Zambia) *“…this time, because you know that you are retaking the course, you take it seriously. Because you feel that itch in your…I don’t have to fail this again. Because at the end of the day, if you fail the retake exam, which means you will be withdrawn from the university…it really makes you work extra hard, or you ask a lot…on the things I didn’t understand, or which I felt like I missed.”* 25 year male MBChB student from uganda

This demonstrates that, while retakes can be emotionally challenging, they also offer opportunities for learning and improvement if students receive adequate support.

### 5. Challenges in Retake Preparation

Students identified several **challenges in preparing for retakes**, including **time constraints, uncertaintyabout the exam content having to adapt to the new environment, the shame**, and **logistical difficulties**.

Students explained how the short time frame between learning about the retake and the exam date affected their preparation:

“…*We don’t always have enough time between learning that we failed and the retake date to properly prepare…*” (31 year male MBChB student from Zambia) Students also noted the challenge of having to encounter a new environment with new students “…*the new environment, like, the new exposure. I was used to my old class… I had to make friends with new classmates, and new faces. It was hard at the beginning… improve as you went along with the retake… the cost of paying fees again was paining”* (30 year male MBChB student from Zambia)

Additionally, students expressed being uncomforted by perceived attitude of lecturers during lectures as they become noted out

*“…lecturer side, well, they could not tell us, but whenever they come, they see you, they would be surprised. I remember this other lecturer, she didn’t want me to speak it out, but she didn’t expect to see me among the retake students…* (28 year male Nursing student from Malawi) Students also noted some devastating statements when retaking *“…we’re referred to as a group of failures, which was really, really frustrating…”* (26 year male Nursing student from Malawi). *“…a retake is a burden to yourself, to the department and your parents…”* 28 year male Nursing student from Malawi. This was compounded by the shame attached as noted by one student *“…the shame I had to encompass while doing this…”* (23 year female nursing student from Nigeria)

Additionally, students faced **financial burdens** related to retakes. One participant highlighted the costs associated with preparing for a retake, particularly for students who had to pay for accommodation and other expenses:

“…*retakes mean extra costs for accommodation, transport, and meals, and that’s very stressful when you don’t have the funds.*” *…. I had to pay fees again, it was not easy* (29 year female MBChB student from Zambia)

This financial strain further compounded the stress associated with retakes, especially for students from economically disadvantaged backgrounds.

### 6. Support Systems

Participants reported that the **support systems** available during the retake process were often inadequate. While **peer and family support** were commonly cited as helpful, many students felt that universities did not provide sufficient **academic or emotional support**.

students noted: “…*My family encouraged me to keep going, but I didn’t get much help from the university…*” (23 year female nursing student from Nigeria) another student also said “…I *got the support from a friend, not the supervisors, because when they just tell you that you have failed, you have to repeat this…”* (31 year male MBChB student from Zambia) *“…I approached some SHO lecturers who were also trying to support me…”* (26 year male MBChB student from Ghana)

Many students suggested that universities should establish **dedicated offices** for students who are struggling academically, where they could receive feedback and guidance. One student emphasized the importance of formal academic support:

“…*If there was an office where students could go after failing to discuss what went wrong, it would help a lot in preparing for the retake…*” (39 year male nursing student from Ghana)

This suggestion reflects a broader need for universities to provide more structured support to students facing retakes.

### 7. Suggestions for Improvement

Students offered several **suggestions for improving the retake process**.

**Table:** 

Suggestions for Improvement	Feedback provision	Need for detailed feedback

	Standardization of assessments	More consistent and relevant assessments
	Repeat only what was failed	Only retake the specific components that were failed
	Mental health resources	Counseling services, emotional support
	Flexible retake schedules	Early announcements, more time for preparation

- **Timely Feedback:** One of the most common suggestions was the need for **detailed feedback** after a failed assessment. As one student put it:

“…*If we know exactly what we did wrong, we can focus on those areas in our preparation…*” (21 year female Nursing student from Nigeria)

- **Standardization of Assessments:** Students called for more **standardized assessments** to ensure fairness. One student recommended:

“…*Only doctors who taught us should be involved in examining us… this would ensure that the exam is based on what we actually learned…*” (25 year female Nursing student from Uganda) Another student also suggested having two or more assessors on a station with further preference of having CCTV cameras *“…having two or three assessors in one station will help, because if you encounter someone who is not in good terms with you, then the other person should be able to back you up. If they cannot manage to do that, at least they should have maybe CCTVs where they can be able to see how the students are being handled…”* (25 year female Nursing student from Uganda)

- **Repeat Only What Has Been Failed:** Some participants suggested that students should only be required to **retake the specific components they failed**, rather than repeating entire courses. One student noted:

“…*I would feel that I should only repeat what I have failed… it makes more sense than repeating the whole course…*” (38 year male MBChB student from uganda) Another student suggested giving extra tasks to help cover the identified lacking skill *“…maybe give me an extra task, but I have moved on to the next level, but give me an extra task…if only OSCE was supplimetable”* (31 year male MBChB student from Zambia)

- **Institutional Support**: Many students emphasized the need for **structured academic, counseling and emotional support** during the retake process. One student suggested:

“…*Universities should provide more structured academic support during the retake process, including feedback and tutoring…*” (22 year female MBChB student from Ghana)

- **Mental Health Resources**: Given the emotional toll of retakes, students suggested that universities should offer **counseling services** to help students manage the stress. One student noted:

“…*Failing an exam can push students into depression; having someone to talk to would make a big difference…*” (38 year male MBChB student from uganda)

- **Flexible Retake Schedules**: Students also recommended **more flexible retake schedules** to allow sufficient time for preparation. One student suggested:

“…*Retakes should be scheduled earlier and results released faster so we can plan better for the next academic year…*” (30 year male Nursing student from Malawi)

### 9. External Factors Affecting Performance

In addition to internal challenges, students identified several **external factors** that contributed to their need for a retake. These included **limited access to instructors during clinical rotations** and **variations in exam difficulty** between different groups

**Table:** 

External Factors Affecting Performance	Limited access to instructors	Inconsistent clinical supervision
	Variability in exam difficulty	Different exam difficulty across student groups

One student shared: “…*some doctors don’t take time to teach us, and then they’re the same ones examining us, asking about things we never covered…*” (22 year female Nursing student from Nigeria)

The inconsistency in exam content across different groups further exacerbated the sense of unfairness in the assessment process. Another student noted:

“…*one group might get an easy set of questions, while another gets a harder set… it’s not fair…*” (26 year male MBChB student from Ghana)

Qualitative findings from in-depth interviews with final-year health profession students across Sub-Saharan Africa who experienced retakes highlight the severe emotional, psychological, and systemic difficulties characteristic of the process of retakes. Many students reported feelings of being shocked, anxious, and fearful; some even mentioned suicidal ideation. These struggles were further compounded by systemic issues-specifically, perceived unfairness of the OSCEs-by lack of feedback that left students uncertain about how to improve. Coping strategies differ: whereas some were supported by their peers and family, others find relief from personal activities such as music. By contrast, formal institutional support was often lacking, with many feeling that they had to deal with their retake preparation in isolation. The experience also had a strong impact on students’ self-perception and motivation: feelings of demoralization, and questioning one’s ability made many feel like giving up. As much as some were willing to take this as an opportunity to learn academically, a financial burden imposed by such a retake, added to its stigma, contributed to their stress. Suggestions from students themselves for improvement ranged from timely feedback and standardized assessments with support systems in place to mental health resources and flexible schedules.

## Discussion

The study aimed to assess the Impact and copying mechanisms of final year nursing and medical students with retakes across five universities in Sub-Saharan Africa towards retakes. The findings revealed psychological and emotional impact as well as various copying mechanisms plus parental and peer support with noted lack of institutional support. This discussion critically interprets these findings and contextualizes them within the broader literature on medical and health professions education.

### Emotional and Psychological Impact of Failure

The qualitative analysis of the emotional and psychological impact of academic failure on medical students reveals profound effects, including feelings of shock, disbelief, fear, anxiety and suicidal thoughts. These reactions are consistent with existing literature that highlights the psychological toll of academic failure, especially in high-stakes environments like medical education. The immense pressure to succeed, coupled with fears of financial and social repercussions, contributes significantly to the emotional burden experienced by students.

Research indicates that academic failure is closely linked to increased levels of stress and burnout among medical students. For instance, studies by Aryaei Moghadam et al., 2023b; Fares et al., 2016; Shadid et al., 2020 confirm that such failures can lead to heightened stress levels, which in turn exacerbate feelings of burnout and negatively impact academic performance and overall well-being.

Moreover, the emotional consequences of failure can diminish self-esteem and motivation which was seen amongst most of the students during their responses, this creates a vicious cycle that further complicates academic challenges. However, some students exhibited resilience and acceptance of the retakes, a phenomenon that aligns with the concept of academic resilience discussed by Aryaei Moghadam et al., 2023b; Fares et al., 2016; Shadid et al., 2020.In summary, the interplay between academic failure, stress, and emotional health is critical in understanding the experiences of medical students. The findings underscore the necessity for supportive measures within educational institutions to help mitigate these adverse effects and promote resilience among students.

This study highlights the urgent need to improve communication between assessors and students, particularly in the context of delivering difficult news, such as retakes, in a manner that minimizes adverse outcomes like severe emotional distress or even suicide. While the medical field has long developed strategies for effectively communicating bad news to patients, such as delivering difficult diagnoses and even death, there remains a significant gap in formalized communication protocols within healthcare teams. Specifically, there is a lack of established guidelines for communication between health workers and between lecturers or instructors and students. This gap is particularly concerning when communicating academic setbacks, such as retakes, where ineffective communication can lead to serious psychological consequences. Developing clear, compassionate, and constructive communication strategies in these educational and professional settings is crucial to prevent potential negative outcomes and to support the overall well-being of students and healthcare professionals.

### Challenges

Another major theme from the qualitative data was the perception of unfairness in the assessment process. Students reported feeling that assessors were biased or rushed, which they believed negatively impacted their performance. These concerns reflect broader systemic challenges in the assessment of clinical skills, where subjectivity and variability in examiner behavior can affect the fairness of evaluations.

Ensuring fairness and equity in clinical assessments, is crucial to accurately evaluating student performance and promoting the development of competent, caring physicians. To achieve this, medical education programs should standardize administration of assessments, ensure transparency in assessment criteria and feedback, utilize multiple examiners per station or case (long or short) to reduce individual biases, blueprint content to the curriculum, and implement quality assurance measures such as examiner calibration and post-exam result review. By adhering to these principles, programs can minimize variability in student performance due to inconsistencies in assessment administration and examiner biases, promoting fairness and equity in clinical assessments and supporting the preparation of students to provide high-quality, equitable patient care, these align with the views of Colbert et al., 2017; Valentine et al., 2023.

### Coping Mechanisms and Peer Support

Despite the significant challenges associated with academic failure and retakes, students employed various coping mechanisms to manage their stress. Acceptance of failure and a focus on improvement were common strategies, with many students turning to peer and parental support for guidance and encouragement. Peer and parental support emerged as a critical resource for students, particularly in the absence of institutional support.

This finding aligns with the work of White et al., 2020, who identified peer support as a key factor in student resilience and success in medical education. In environments where formal academic support is lacking, peer networks can provide emotional and academic assistance, helping students navigate the retake process more effectively.

## Conclusion

This study gives critical insights into the retake proportions among final-year medical and nursing students across five universities in Sub-Saharan Africa.

The investigation stated that students who had to retake classes suffered from severe emotional and psychological impacts, often heightened by a lack of institutional support and perceived unfairness in assessment. Detailed feedback after a retake was not provided, further hindering the students from learning and improving. Support from peers and family members was an important source in dealing with such situations, while formal academic guidance was negligible.

Such findings bring into focus the implementation of targeted interventions by the institutions to improve the feedback mechanisms, make transparent assessment processes, and put in place comprehensive support systems-both academic and psychological-in order to minimize the occurrences of such retakes and thereby achieve excellence in the student performance in health professions education in the region. Further research is required to see how such a model of retakes influences the long-term professional development and to develop effective remediation strategies.

## Supplementary Material

Supplement 1

## Figures and Tables

**Figure 1:**
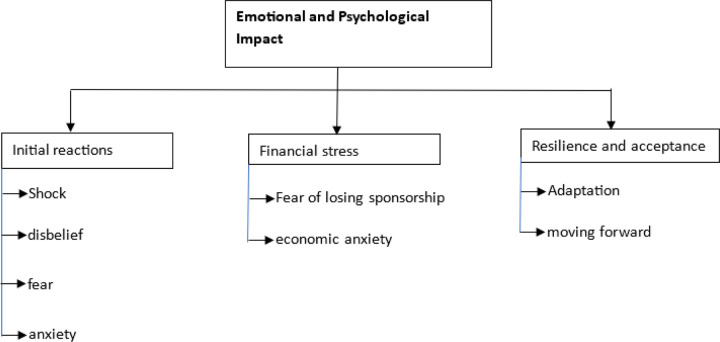
Unnumbered image in the Results section.

**Figure 2:**
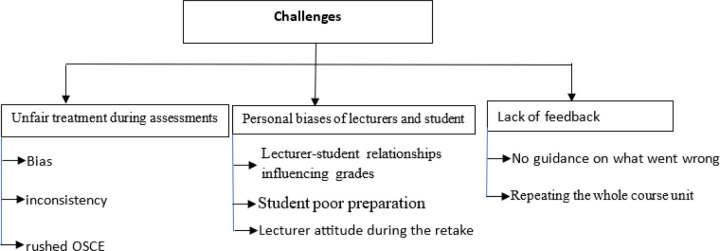
Unnumbered image in the Results section.

**Figure 3:**
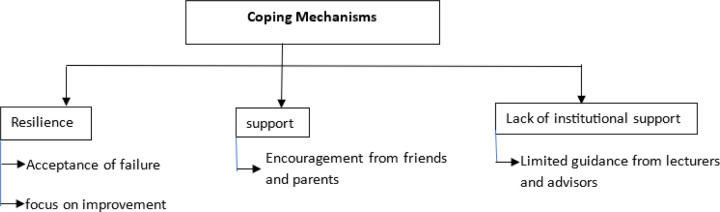
Unnumbered image in the Results section.

**Figure 4:**
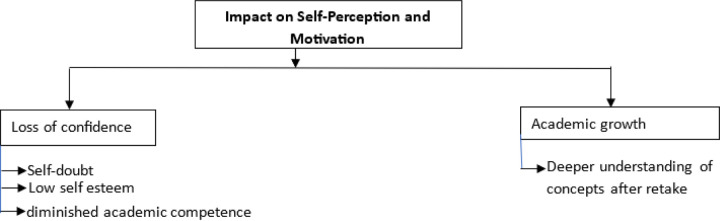
Unnumbered image in the Results section.

**Figure 5:**
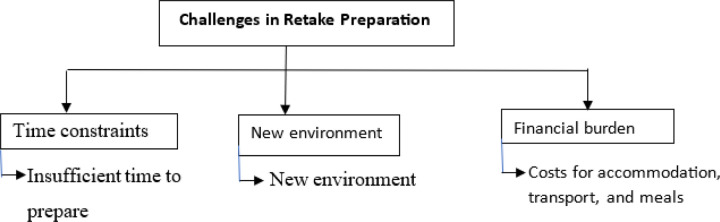
Unnumbered image in the Results section.

**Figure 6:**
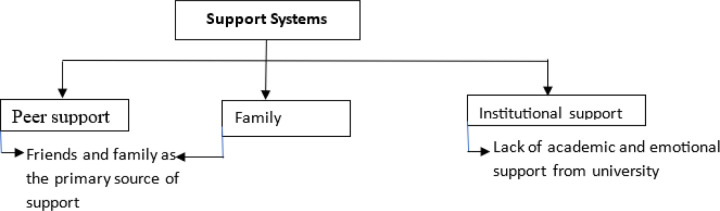
Unnumbered image in the Results section.

**Table 1:** sample size estimates

University	totals population	calculated sample size
Busitema University from Uganda	85	70
University of Ibadan from Nigeria	184	125
Kwame Nkrumah University of of science and technology, Kumasi from Ghana	132	99
Mzuzu University from Malawi	244	142
University of Zambia from Zambia	206	135
	851	571

**Table 2:** Demographic characteristic of the Study Particpants

Variable	Category	Frequency	Percent (%)
Gender	Male	287	37.6
	Female	473	61.9
	Prefer not to Say	4	0.5
Religion	Islam	44	5.8
	Christian	691	90.4
	Ashanti	13	1.7
	Not religious	7	0.9
	Not specified	9	1.2
Variable	N	Mean ± SD	Range
Age (years)	764	25.38 ± 5.00	18–48
Variable	Category	Frequency	Percent (%)
Age group (years)	18–24	416	54.5
	25–34	292	38.2
	35 and above	56	7.3
Program	MBcHB	271	35.5
	Nursing	493	64.5
Level of Program	Bachelor’s Degree	764	100.0
Level of Entry	Extensor/Upgrading	118	15.4
	Direct	500	65.4
	Unified Tertiary Matriculation Examination (UTME)	137	17.9
	others	9	1.2
University	Busitema University from Uganda	85	11.1
	University of Ibadan from Nigeria	135	17.7
	Kwame Nkrumah University of of science and technology, Kumasi from Ghana	198	25.9
	Mzuzu University from Malawi	195	25.5
	University of Zambia from Zambia	151	19.8

**Table 3:** proportion of students who have ever experienced a retake in clinical evaluation

Have you ever failed a paper in your clinical years?						
		yes		No		
	Frequency		(%)	Frequency	(%)	P-value*
Busitema University from Uganda		10	12	73	88	< 0.001
Mzuzu University from Malawi		43	22.1	152	77.9	< 0.001
University of Ibadan from Nigeria		8	5.9	127	94.1	< 0.001
Kwame Nkrumah University of of science and technology, Kumasi from Ghana.		3	2.5	117	97.5	< 0.001
University of Zambia from Zambia		22	14.7	128	85.3	< 0.001
Overall		86	12.6	597	87.4	< 0.001
