# Supplementary material for: Impact and copying mechanisms towards retakes: A case study of five health training institutions in Sub Saharan Africa
Source: Res Sq. 2024 Dec 16:rs.3.rs-5374432. Preprint. [Version 1] doi: 10.21203/rs.3.rs-5374432/v1 (PMC11702798; doi:10.21203/rs.3.rs-5374432/v1)
Supplement: Supplement 1 [file NIHPPRS5374432v1-supplement-1.pdf]

Supplementary Files

This is a list of supplementary files associated with this preprint. Click to download.

- additionaltablesforimpactandcopyingmechanisms.docx
